# Supplementary figures and images for: Cryptococcus neoformans Slu7 ensures nuclear positioning during mitotic progression through RNA splicing
Source: PLoS Genet. 2024 May 20;20(5):e1011272. doi: 10.1371/journal.pgen.1011272 (PMC11142667; doi:10.1371/journal.pgen.1011272)

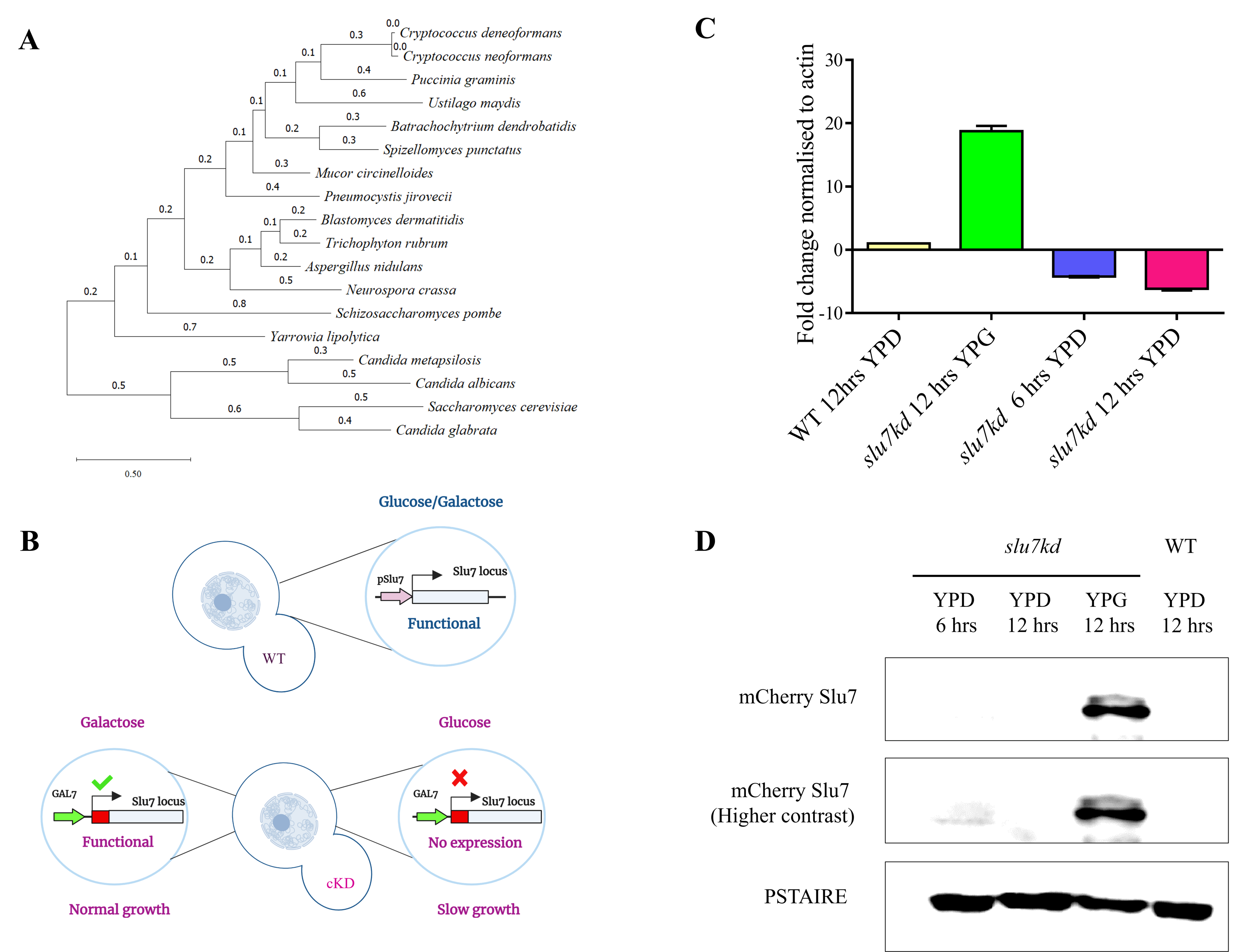

Supplement: S1 Fig — (A) Phylogenetic tree conservation of Slu7 protein among fungi group. The tree was generated using MEGA 11 with Maximum Likelihood method. The branch lengths measured in the number of substitutions per site is denoted above the branches. (B) Schematic representation of Slu7 knockdown strain. (C) Quantification of RNA depletion in slu7kd and wildtype cells after the shift into non-permissive media for 6 hours and 12 hours by qRT PCR. (D) Western blot to detect mCherry tagged Slu7 protein in slu7kd grown in non-permissive media for 6 hours and 12 hours at 30°C. The blot was probed with anti-mCherry as described in the Materials and Method section. pSTAIRE was used as loading control. Knockdown strain grown in permissive media for 12 hours was used as positive control. (TIF) [file pgen.1011272.s001.tif]

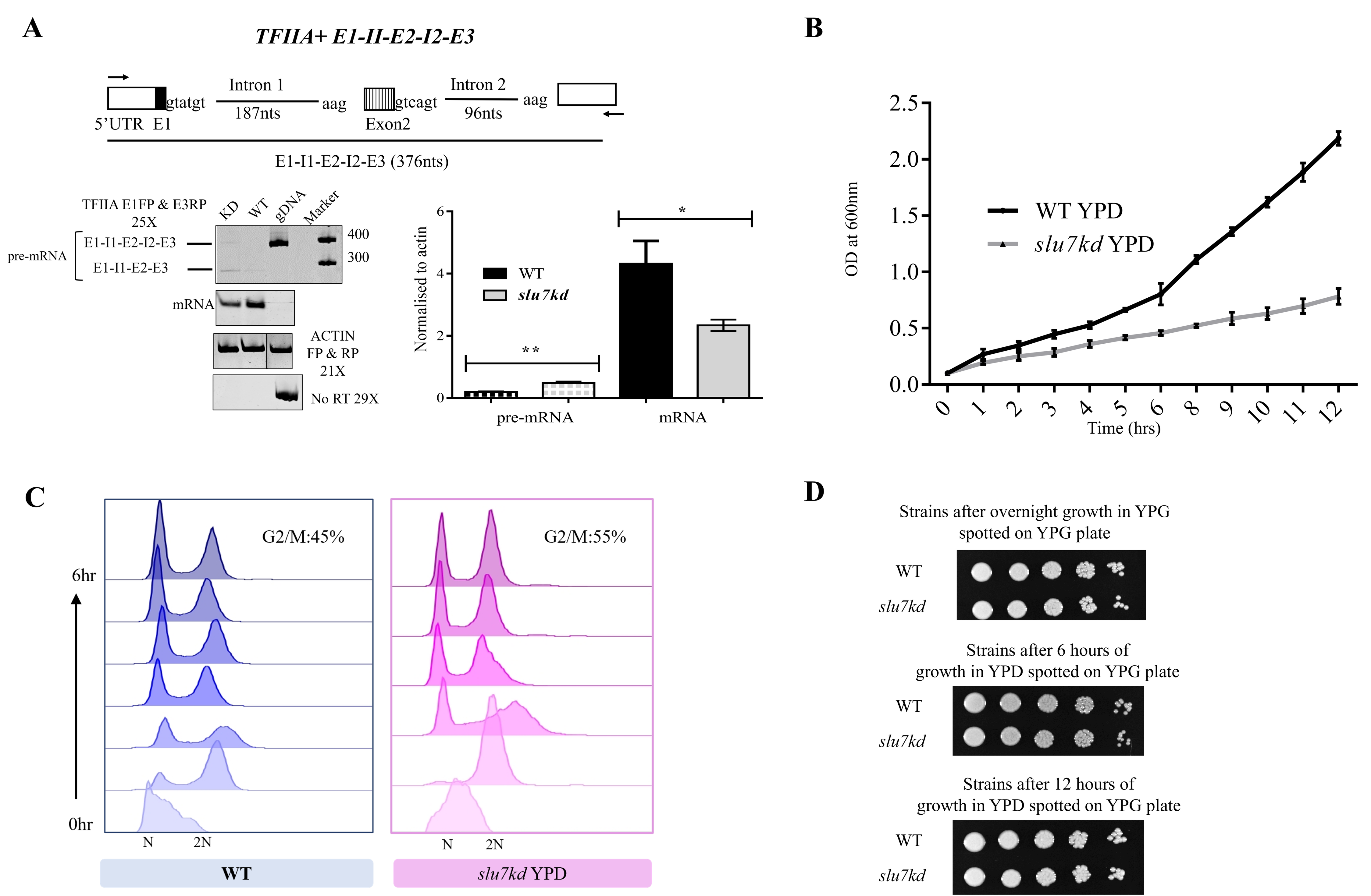

Supplement: S2 Fig — (A) Splicing defect of TFIIA intron 1 in slu7kd and wildtype grown in YPD for 12 hours. The reactions marked as “No RT 29 X” denote semi- quantitative PCR performed on DNase treated RNA sample, without reverse transcription of RNA to cDNA. (B) Growth profile of broth cultures of wildtype and slu7kd grown in non-permissive media. The data represent mean ± SD for three independent biological replicates. (C) Flow cytometry analysis of synchronous wildtype and Slu7 conditional knockdown after release into non-permissive media. The 0th time point indicates the HU arrested cell population before the release into permissive media. The percentage at the top represents the % of cells in the G2/M phase at the end of 6 hours, N = 3. (D) Serial 10-fold dilution of 2 X 105 cells from wildtype, slu7kd strain. The strains were grown in non–permissive media for 6 hours and 12 hours and spotted on permissive media and monitored for growth at 30°C for 5 days to assess the loss of viability. (TIF) [file pgen.1011272.s002.tif]

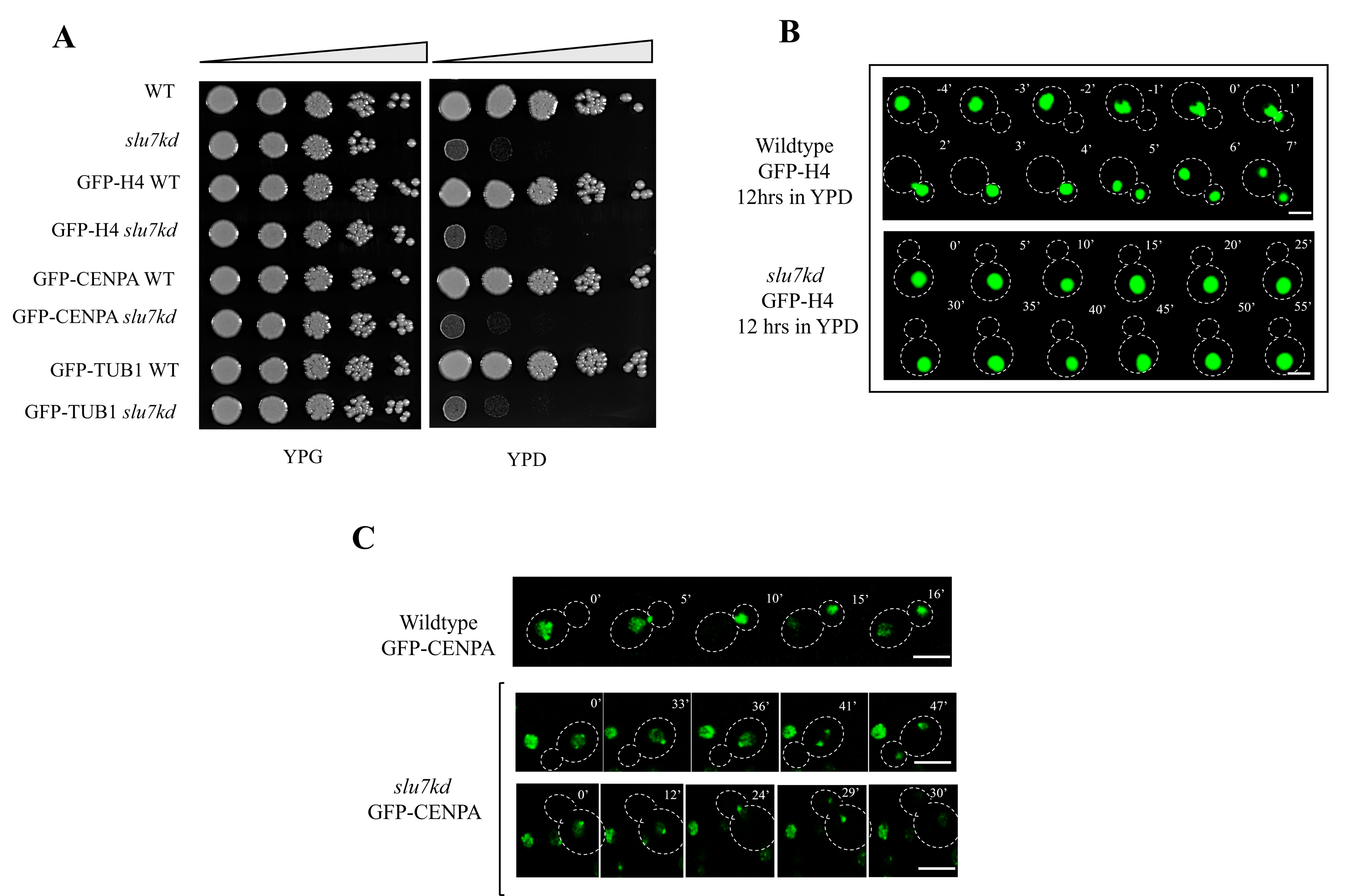

Supplement: S3 Fig — (A) Serial 10-fold dilution of 2 X 105 cells from wildtype, slu7kd strain, each of which had marked reporters for monitoring mitosis. These strains with GFP-H4, GFP-TUB1, and GFP-CENPA reporters were spotted on non-permissive media and monitored for growth at 30°C for 5 days. (B) Time-lapse snapshots of slu7kd and wildtype cells with GFP-H4 reporter to visualize nuclear dynamics after growth in non-permissive media for 12 hours. T = 0 was taken when the nucleus enters the neck region in the wildtype panel. In the knockdown panel, the timestamps are mentioned right from the start of the imaging. Bar, 5μm. (C) Time-lapse snapshots of slu7kd and wildtype cells with GFP-CENPA reporter to visualize the kinetochore dynamics after growth in non-permissive media for 6 hours. T = 0 represents the start of the live imaging. Bar, 5μm. (TIF) [file pgen.1011272.s003.tif]

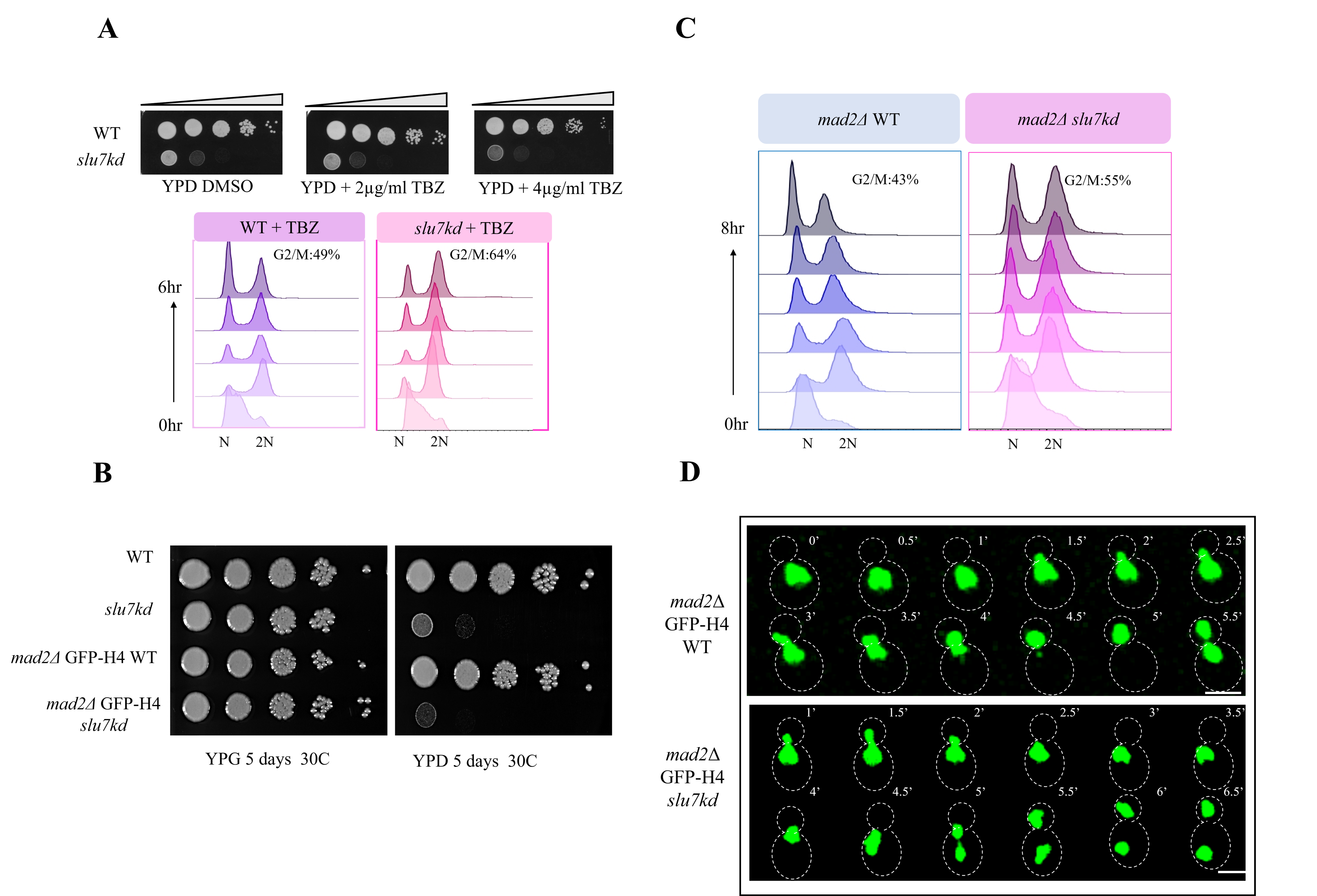

Supplement: S4 Fig — (A) Serial 10-fold dilution of 2 X 105 cells from wildtype and slu7kd spotted on non-permissive media containing 2μg/ml and 4μg/ml thiabendazole and monitored for growth at 30°C for 5 days. Flow cytometry analysis of cells from wildtype and slu7kd strain withdrawn at various time points after inoculation of HU- synchronised cells into non-permissive media containing 4μg/ml thiabendazole. The percentage figures given at the top represents the % of cells in the G2/M phase at the end of 6 hours, N = 3. (B) Serial 10-fold dilution of 2 X 105 cells from wildtype and slu7kd in the background of mad2Δ GFP-H4 spotted on non-permissive media and monitored for growth at 30°C for 5 days. (C) Flow cytometry analysis of cells from wildtype and slu7kd strain in the background of mad2Δ GFP-H4 withdrawn at various time points after inoculation of HU- synchronised cells into non-permissive media. The percentage figures given at the top represents the % of cells in the G2/M phase at the end of 8 hours, N = 3. (D) Time-lapse snapshots of slu7kd and wildtype cells in the background of mad2Δ GFP-H4 to visualize nuclear dynamics after growth in non-permissive media for 6 hours. T = 0 was taken when the nucleus enters the neck region between the mother and daughter cell. Bar, 5μm. (TIF) [file pgen.1011272.s004.tif]

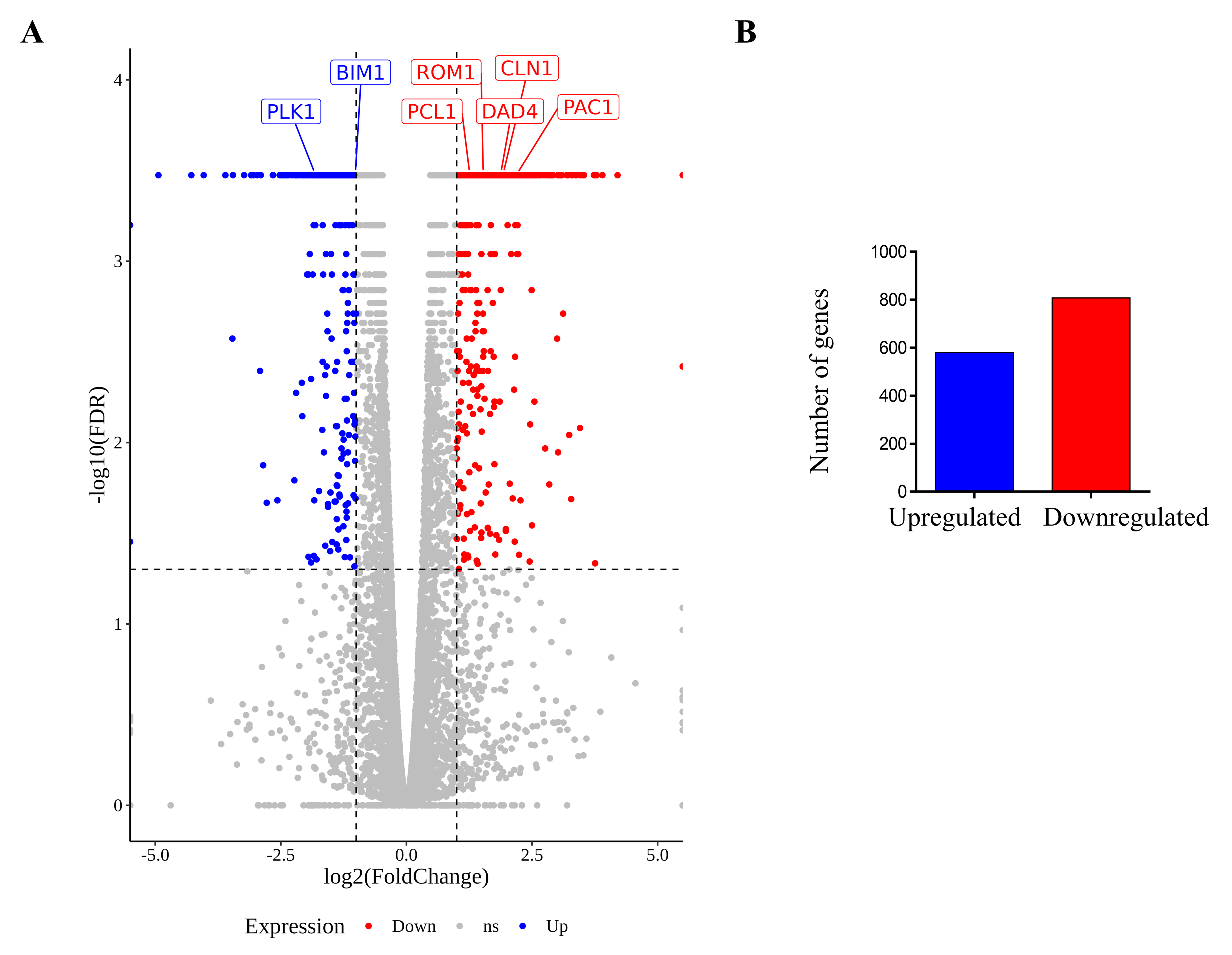

Supplement: S5 Fig — (A) Volcano plot representing the differential gene expression between slu7kd and Wildtype. The dotted line parallel to X-axis denotes the cutoff for -log10(FDR), of 1.303 (FDR value less than 0.05), and the dotted lines parallel to Y-axis denote the cutoff for log2(fold change) of –1 or +1. (B) The bar chart represents the upregulated and downregulated genes in Slu7 knockdown compared to wildtype. (TIF) [file pgen.1011272.s005.tif]

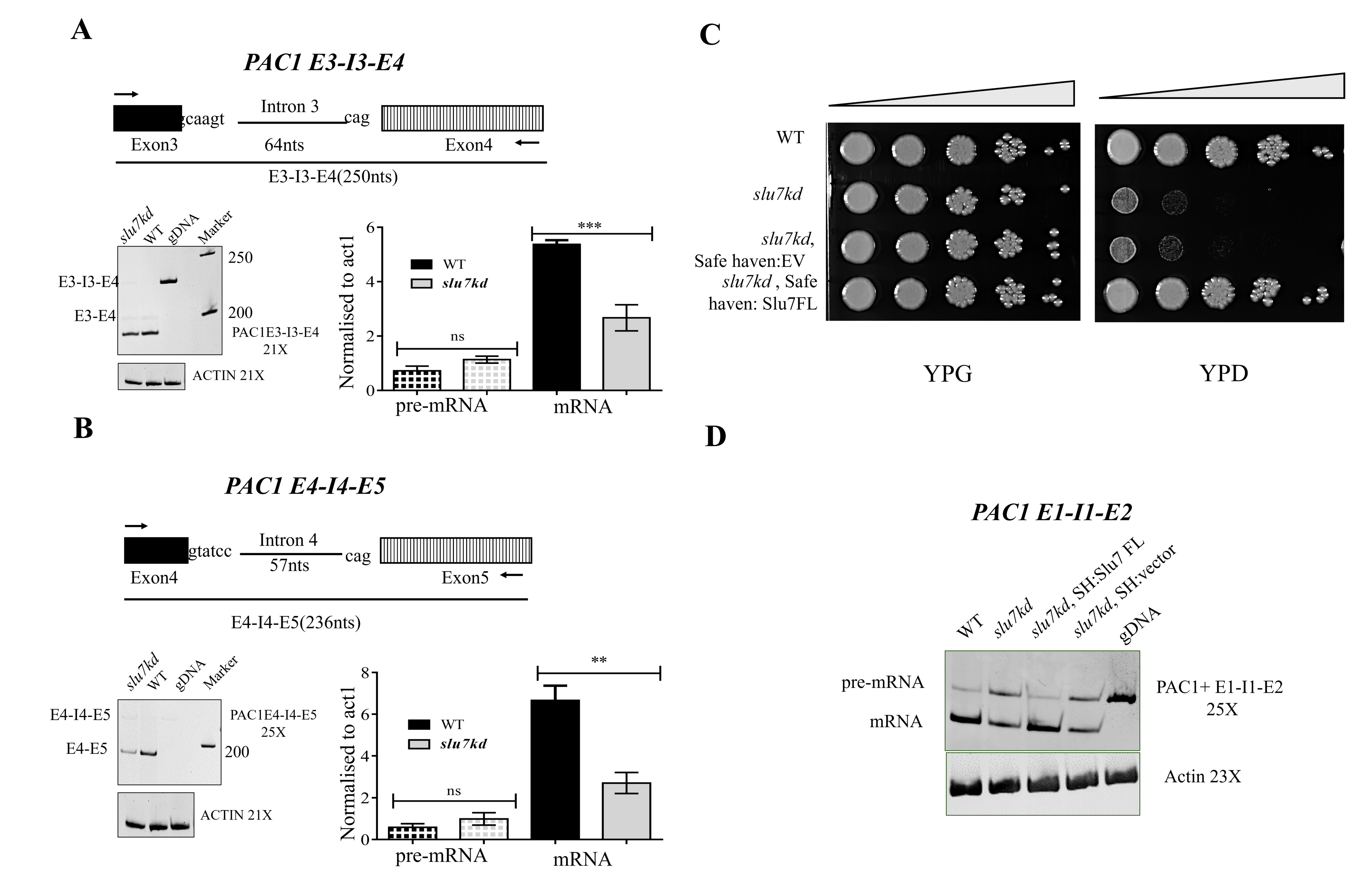

Supplement: S6 Fig — Schematic representations show each intron together with its flanking exons. Intron length is given within brackets. RNA from WT and slu7kd cells grown at 30°C for 12 hours was taken for limiting cycle, semi-quantitative RT-PCR using the flanking exonic primers. For each intron, the pre-mRNA (P) or mRNA (M) levels were normalized to that of the ACT1 mRNA. The normalized pre-mRNA or mRNA levels are plotted. The data represent mean ± SD for three independent biological replicates. p values were determined by unpaired Student’s t-test. ns, non-significant change with p > 0.05. (A) The splicing status of the PAC1 intron 3 in wildtype and slu7kd. (B) The splicing status of the PAC1 intron 4 in wildtype and slu7kd. (C) Serial 10 dilutions starting from 2 X 105 cells of wildtype, Slu7 conditional knockdown, and two transformants expressing Slu7FL from safe haven locus in the background of slu7kd were spotted on non-permissive media. The image was obtained after incubating the plates at 30°C for 5 days. (D) The splicing status of the PAC1 intron 1 in wildtype, slu7kd, and slu7kd expressing Slu7FL from safe haven locus. RNA from WT, slu7kd, and slu7kd cells expressing Slu7FL from safe haven locus grown at 30°C for 12 hours was taken for limiting cycle, semi-quantitative RT-PCR usinssg the flanking exonic primers. The experiment was done in three independent biological replicates. (TIF) [file pgen.1011272.s006.tif]

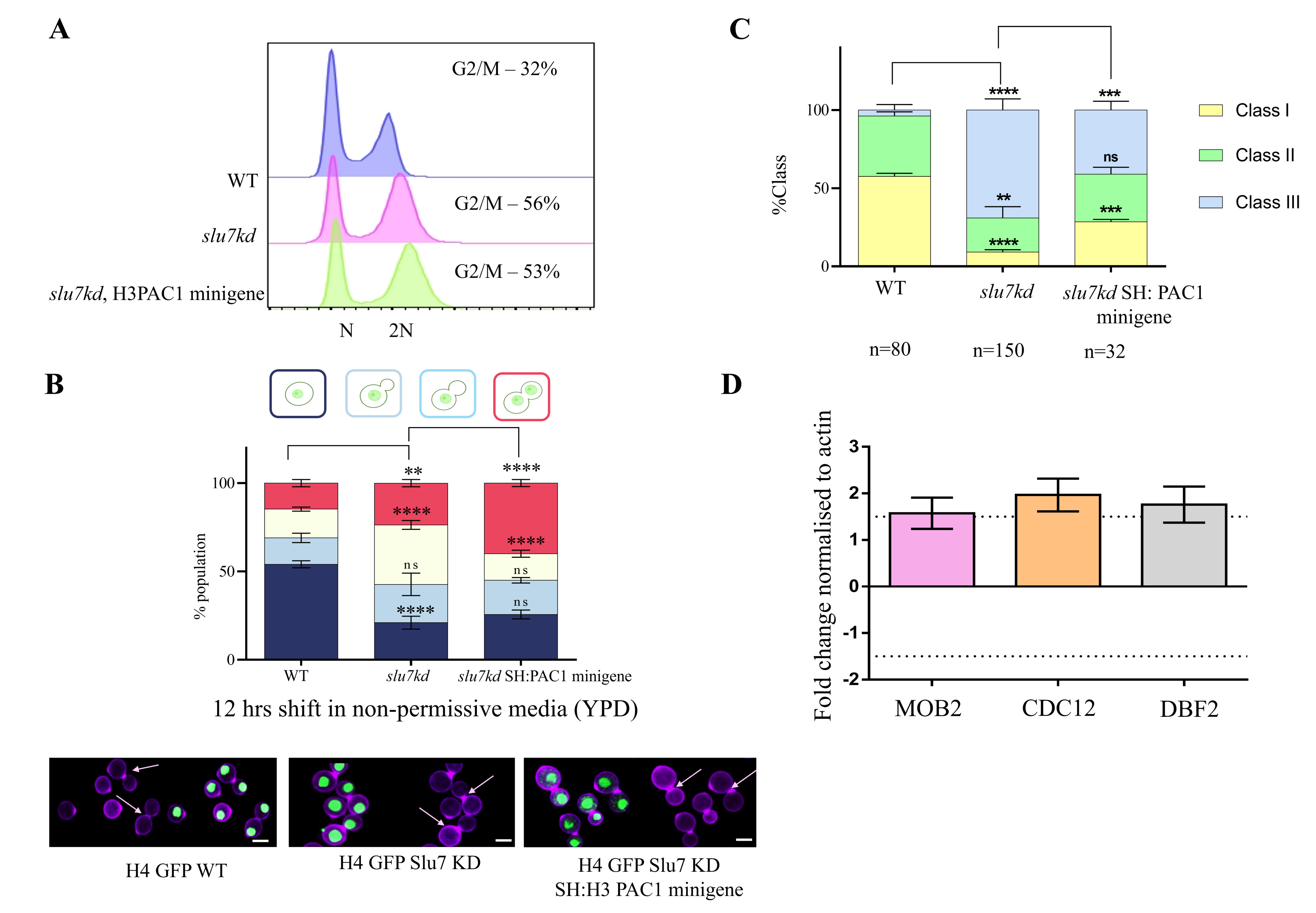

Supplement: S7 Fig — (A) Flow cytometry analysis of cells from wildtype, slu7kd and slu7kd expressing PAC1 minigene grown in non-permissive media (YPD) for 12 hours. (B) The percentage of cells at various phases of the cell cycle, based on bud and nuclear position was measured using slu7kd (n = 100), wildtype (n = 100) and slu7kd SH::PAC1 minigene (n = 100) was measured in fixed cells (4% paraformaldehyde) after growth in YPD for 12hrs, respectively. The data represent mean ± SD for three independent biological replicates. One-way ANOVA test followed by Turkey’s multiple comparison test was used to calculate the statistical significance of differences between the population (the p values show the difference compared to the wildtype vs slu7kd, slu7kd vs slu7kd SH:PAC1 minigene). Snapshots of wildtype GFP-H4 WT, slu7kd GFP-H4, and slu7kd with H3:PAC1intronless minigene GFP-H4 cells stained with calcofluor white to visualize the cell wall. Bar, 5μm. (C) Localization of Dyn1 in the wildtype, slu7kd and slu7kd with PAC1 minigene cells at different stages expressing Dyn1-3xGFP upon their growth in the non-permissive conditions. Percentages of cells with different pattern of dynein signal are quantitatively represented in the bar graph. The yellow bar represents cells with clustered dynein puncta both in mother and daughter bud in large budded cells. The green bar represents cells with multiple dynein puncta only in the mother bud of large-budded cells, and blue bar shows % cells with no dynein puncta either the mother and daughter bud of large budded cells. The data represent mean ± SD for three independent biological replicates with n ≥ 34 large-budded cells. Bar, 5μm. This experiment comes with a technical limitation to capture the nuclear position with respect to dynein position. The low sample size (metaphase cells) in slu7kd overexpressing PAC1 minigene is because high proportion of cells arrested post mitosis. (D) qRT-PCR to assess the deregulation of cytokinesis-related genes in knockd [file pgen.1011272.s007.tif]
