# Supplementary material for: Cryptococcus neoformans Slu7 ensures nuclear positioning during mitotic progression through RNA splicing
Source: PLoS Genet. 2024 May 20;20(5):e1011272. doi: 10.1371/journal.pgen.1011272 (PMC11142667; doi:10.1371/journal.pgen.1011272)
Supplement: Attachment — Submitted filename: 142023_1_rebuttal_2951091_s9kcrd.pdf [file pgen.1011272.s012.pdf]

Full Revision

Review COMMONS

Manuscript number: RC-2023-02270
Corresponding author(s): Usha Vijayraghavan

General Statements

We thank all three Reviewers for their thorough assessment of our manuscript and their constructive feedback and comments.

Point-by-point description of the revisions

This section is mandatory. Please insert a point-by-point reply describing the revisions that were already carried out and included in the transferred manuscript.

Reviewer #1

We are encouraged by the very positive comments made on the significance of our study that it provides convincing insights on alternative modes of nuclear positioning and division which is an important question in cell biology. We also took all possible suggestions to improve the interpretation of our results, have also added some newer data to address the constructive points raised by the reviewer.

Major comments:

1. A) I am concerned about the lethal phenotype caused by slu7 deprivation. Slu7 deficiency causes defective nuclear positioning at the bud in late G2. This phenotype per se should not cause defective mitosis, so slu7 deficiency may also be interfering with other aspects of mitosis which might indeed impinge on cell viability.

Response: Our data indeed show Slu7 knockdown has severe growth defect when grown on non-permissive media (YPD) where a two-fold difference in O.D. was seen by 12 hours (Supplementary figure 2.B).
We agree with the reviewer that defective mitosis, arises from several aspects of cell cycle including those in mitosis. The data we present show G2 arrest, small-budded cells with unsegregated nuclei and large-budded cells with segregated nuclei, all which do not progress through cell cycle phases and contribute to the severe growth defect. Further, GO enrichment analysis of deregulated pathways on knockdown of Slu7 support the above findings as various cell cycle related pathways are abnormal in their expression levels. In this study, we have focused on an in depth analysis of the role of Slu7 in a particular window and uncover how it

controls nuclear position for progress G2-M phase cell cycle progression. The likely targets and mechanisms by which Slu7 regulates other phases of the cell cycle which needs similar other deeper investigations in future. Our detailed analysis of nuclear movement in Slu7 knockdown cells grown in YPD for 12 hours showed no nuclear movement (Supplementary figure 3B) which is the terminal phenotype. To examine events that lead to nuclear mispositioning phenotype we investigated the dividing *slu7kd* cells grown in non-permissive media for only 6 hours; under these conditions Slu7 protein is still detected at lower amount (Supplementary figure 1D). From the studies of nuclear position, mitotic spindle position and dynein distribution in mother and daughter cell, we propose that in the dividing cells, the nucleus does not experience enough force to move inside the daughter bud during mitosis. Further, we delineate the role of Slu7 in the splicing of transcripts for PAC1 encoding a protein whose homolog in S. cerevisiae has a proven role in nuclear migration. In live imaging of *slu7kd* cells that show nuclear segregation at the start of live imaging, new bud was not formed till the end of 60 minutes, implying that are arrested after transition to mitosis. We could speculate a role for Slu7 through regulation of genes involved in mitotic exit or cytokinesis.

B) Supp. Fig4 shows defective mitosis in TBZ, so TBZ may be exacerbating defective mitosis of slu7-deficient cells.

Response: Studies with yeast and mammalian model systems have revealed that the mobility and repair of damaged DNA are compromised upon disruption of microtubules (Wu et al, 2008; Chung et al, 2015; Lottersberger et al, 2015; Lawrimore et al, 2017; Oshidari et al, 2018; Laflamme et al, 2019). These data point to reasons why the mutants in DNA damage checkpoint genes are sensitive to TBZ. In this context, we observed that CnSlu7 knockdown is also sensitive to MMS stress (shown below). In addition, recent work on human Slu7 in Hela cell lines has elucidated the its role in the maintenance of genome integrity by preventing the formation of R-loops (Jiménez et al, 2019). We suggest that TBZ may exacerbate the defective mitosis of Slu7 depleted cells, however, whether it is particular only to mitosis or to the other cellular processes where the microtubules are involved needs further investigation.

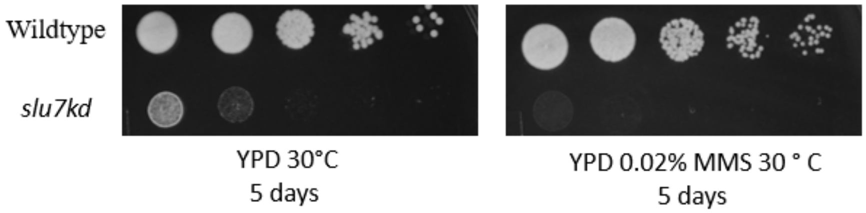

2. Throughout the figures it can be observed uneven chromosome/nuclear segregation in cells deprived of slu7, however, these mitotic defects have not been mentioned or explored in depth. From Supp Figure 3C it can be inferred that CENP-A segregation is uneven. Is this correct? Is CENP-A-GFP segregation normal?

Response: It should be noted that in Cryptococcus, the kinetochore remains unclustered during the early phase of cell cycle, cluster to a single punctum at the end of G2 phase and then de-cluster at the end of mitosis. Since this is a highly dynamic process, its technically challenging to measure the intensity CENP-A in mother and daughter cell. In the fixed cell imaging or live imaging data, there are no appreciable differences in intensity of the GFP signal of the tagged proteins (H4 and CENPA). The uneven chromosome/nuclear segregation observed in certain panels images presented are due to technical issues in that particular stack while generating the montage. This has been re-examined and we infer that there are no major differences in the signals from GFP-H4 and GFP - CENPA through mitosis.

Additionally, taking the cue from the reviewer’s comment, we examined the likelihood of improper chromosome segregation by evaluating if there are any appreciable cell populations that are aneuploid. We revisited our flow cytometry data, we found no significant difference in the population of aneuploid cells between the knockdown strain and wildtype strain grown in non-permissive condition for 12 hours. This data was assessed again in new experiments where we also analyzed by flow cytometry the *ipl1* mutant where aneuploidy is reported (Varshney et al, 2019). It has been reported in Cryptococcus neoformans that aneuploid cells are resistance to anti-fungal drug fluconazole. Preliminary experiments showed that *slu7kd* cells were sensitive to fluconazole and in this assay were similar to wildtype cells. Hence, we speculate that chromosome segregation is normal in Slu7 depleted cells.

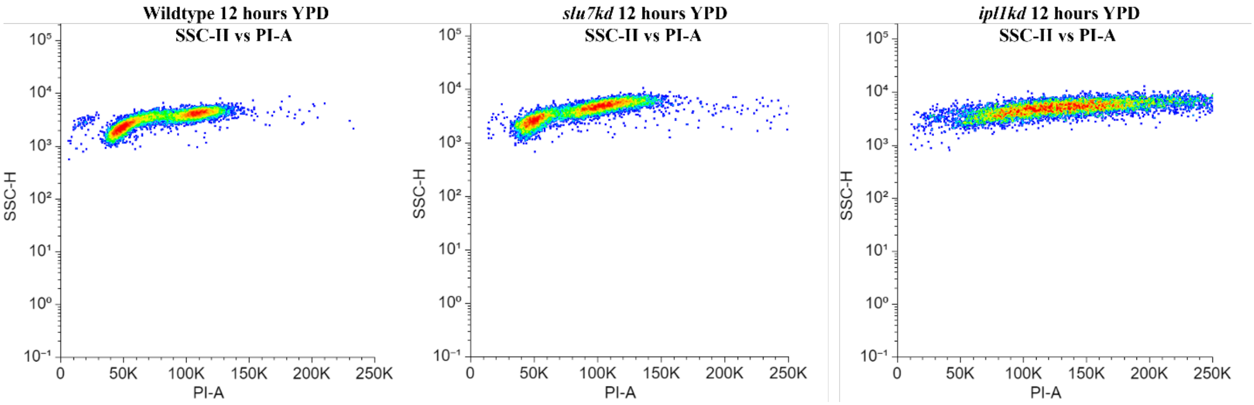

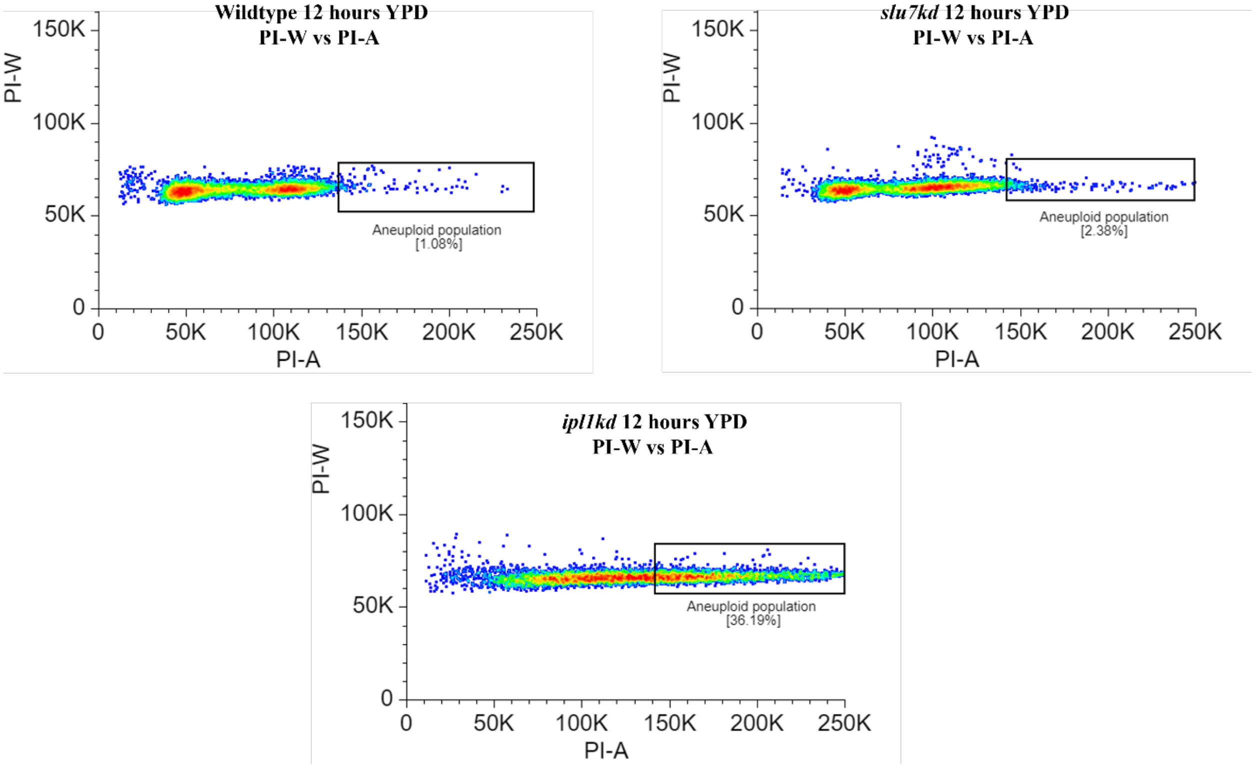
Wildtype 12 hours YPD
PI-W vs PI-A
Aneuploid population [1.08%]
slu7kd 12 hours YPD
PI-W vs PI-A
Aneuploid population [2.38%]
ipl1kd 12 hours YPD
PI-W vs PI-A
Aneuploid population [36.19%]
PI-W
PI-A
150K
100K
50K
0
50K
100K
150K
200K
250K

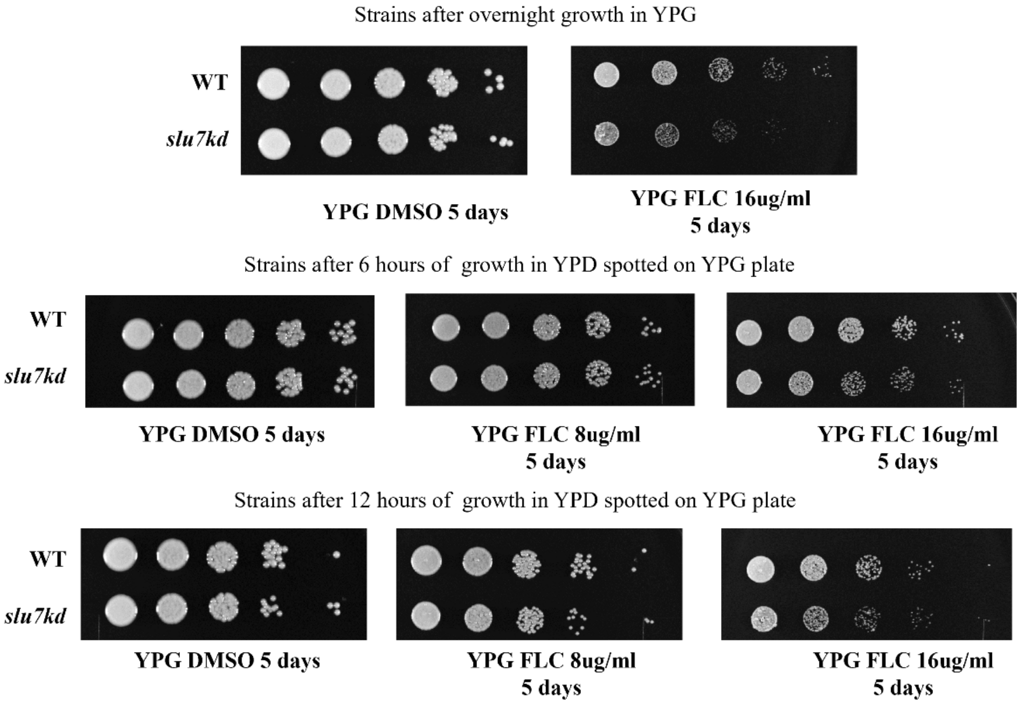
Strains after overnight growth in YPG
WT
slu7kd
YPG DMSO 5 days
YPG FLC 16ug/ml
5 days
Strains after 6 hours of growth in YPD spotted on YPG plate
WT
slu7kd
YPG DMSO 5 days
YPG FLC 8ug/ml
5 days
YPG FLC 16ug/ml
5 days
Strains after 12 hours of growth in YPD spotted on YPG plate
WT
slu7kd
YPG DMSO 5 days
YPG FLC 8ug/ml
5 days
YPG FLC 16ug/ml
5 days

3. If chromosome segregation is altered upon slu7 deprivation, this might also explain the drop in cell viability and slow growth rates of this condition.

Response: From live microscopy imaging and flow cytometry data, we believe that the chromosome segregation is normal in Slu7 depleted cells. Dilution spotting in permissive media after growth in non-permissive media revealed that *slu7kd* cells resumed growth without losing viability, indicating the arrest phenotype associated with the depletion of Slu7 is largely reversible and does not cause chromosome mis-segregation (figure is now added to manuscript as supplementary figure 2D). Prolonged arrest at various cell cycle phase might lead to cell death and hence drop in cell viability.

4. The manuscript will improve if authors analyse chromosome segregation for example, by showing time-lapse images of chromosome dynamics during mitosis.

Response: Chromosome dynamics during the mitotic phase is given below. We observe that the chromosome segregation is equal in both mother and daughter bud. The uneven chromosome/nuclear segregation observed in certain panels images presented in original manuscript were due to technical issues while generating the montage.

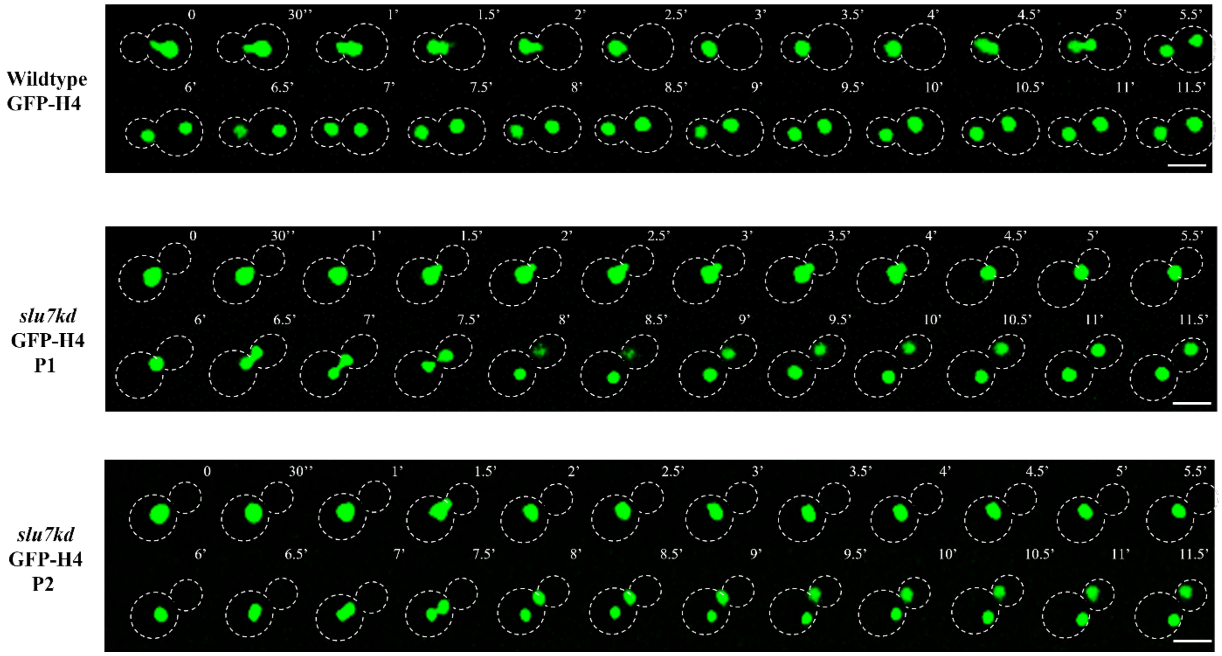

5. The authors perform an RNA seq comparing wild-type cells with slu7 deficiency and detect changes in gene expression, however, they do not explore from this data the percentage of un-spliced introns genome-wide which might be very informative, even more than changes in gene expression, which many of them, might be an indirect consequence of Slu7 deficiency. Authors should re-analyze the RNA seq data looking for unprocessed mRNAs and provide information about the overall impact of slu7 in intron processing.

Response: A very detailed bioinformatic analysis of the impact on slu7 on global transcriptome and splice pattern, is an ongoing study in the laboratory. The findings are indeed giving good leads which are being validated by further experiments using mini-gene exon-intron constructs. These studies are extensive and form a future manuscript identifying and characterizing intronic features which predispose an intron towards Slu7 dependency. Therefore, it falls outside the scope for this study on the cell biological role of Slu7 on mitosis, specifically nuclear position to ensure faithful mitotic segregation.

Minor comments:

1. "Previous studies of slu7 mutants in S. cerevisiae and the conditional knockdown of its S. pombe homolog". Consider replacing homolog with Ortholog.

Response: The suggestion is well taken, and the word "homolog" has been replaced with word "ortholog".

2. A) Taking these results together, we conclude that the inability of the conditional mutant to grow in the non-permissive media is due to impaired progression through the G2-M phase of the cell cycle.
Is the G2/M delay the cause of the slow growth phenotype of the Slu7 deficiency?

Response: From the live microscopy, we note that even when the budding index for mitosis has been reached the nucleus in *slu7kd* cells is still in the mother cell and spends more time here rather than reaching the bud or bud neck. We present G2/M delay as ONE of the reasons for the slow growth of Slu7 depleted cells. Although we have showed that Slu7 depletion does not activate MAD2 dependent Spindle Assembly Checkpoint, we have not investigated the activation of other cell cycle checkpoints such as G2 DNA damage checkpoint. These are potential new leads as we infer from our RNA seq datasets that CHK1, TEL1, BDR1 and RAD51 show increased expression in Slu7 knockdown condition when compared to wildtype. It is therefore reasonable to conclude that Slu7 might play a role at various cell cycle phases through direct or indirect effect on genes involved in these phases. Delayed positioning of the nucleus during G2/M is one of the major effects that is investigated in depth in this study.

B) If so, growth defects of slu7 deficiency could be suppressed by ectopic expression of G2/M activators.

Response: We have not tested this possibility, but we predict that expression of G2/M activators would at best offer only partial rescue the growth defect of Slu7 depleted cells since multiple pathways are adversely affected in cells depleted of Slu7.
In this line of investigation, we have tested the consequences of PAC1 overexpression, as PAC1 expression levels and splicing are affected by loss of Slu7. We report a partial rescue of nuclear position defect during mitosis, yet these cells were arrested at cytokinesis. Further, the unavailability of an array of suitable auxotrophic (or other) markers in this model system makes it technically challenging to do rescue experiments by overexpression of multiple candidate downstream genes.

3. Supp Figure 3C, remove the drawing on the right. Adjust times relative to panels.

Response: The drawing has been removed and the time points have been adjusted.

4. Tracking the nucleus in wild-type cells with a small bud showed that the nucleus moved into the daughter bud, divided into two, and one-half migrated to the mother bud (Supplementary Figure 3B, top row).

Please replace the sentence: "one-half" with "one of the daughter nuclei".
Additionally, as this nuclear positioning occurring during late mitosis is due to spindle elongation, I would not use the term migrated but "positioned" or "moved". Nuclear movement into the bud, which is referred to as "moved", can indeed be named "migrated".

Response: The word “migrated” in the above sentence has been replaced with the word “moved”.

5. Indicates in Figure 2B the marker used (GFP-H4), as in Fig Supp 3B.

Response: The marker has been indicated in the figure.

6. Nuclear division initiates in the bud, and one of the divided nuclei with segregated chromosomes migrates back to the mother cell (Figure 2B, top panel, wildtype, quantified in Figure 2C grey bar).

As mentioned before, I would not name this, nuclear migration as it is the result of spindle elongation, and it can be confusing or misleading for non-expert readers.

Response: The word "migrate" in the above sentence has been replaced with the word "move".

7. These two conclusions should be revised and described in temporal/sequential order.

1. Thus, we identify that the depletion of CnSlu7 severely affects the temporal and spatial sequence of events during mitosis, particularly nuclear migration and division.

2. Together, these results confirmed that without affecting the kinetochore clustering, depletion of Slu7 affects nuclear migration during the G2 to mitotic transition in Cryptococcus neoformans.

Response: We thank the reviewer for bringing out the clarity in the concluding statements. These has now been revised to read as follows:
"Together, these results confirm that without affecting the kinetochore clustering, depletion of Slu7 affects nuclear movement during the G2 to mitotic transition in Cryptococcus neoformans. Thus, we identify that the depletion of CnSlu7 severely affects the temporal and spatial sequence of events during mitosis, particularly nuclear migration, and division."

8. In slu7d cells, in cells with small buds, numerous cMTs were nucleated from the MTOCs, and as the cell cycle progressed, they organized to form the unipolar mitotic spindle (Figure 3A, slu7kd GFP-TUB1 panel, time point 55 mins).
Please, revise whether the term unipolar mitotic spindle is correct here.

Response: The word unipolar has been removed.

9. I suggest including page and line numbers in the manuscript to facilitate revision.

Response: We regret missing out this formatting guideline. The Page and line numbers have provided.

Reviewer #2

We are thankful by the very positive comments on the significance of our work, its novelty and findings being of broad interest to microbiology; splicing; cell cycle and cell division communities. We respond to all comments raised below.

1. The authors test the Mad2-dependent spindle assembly checkpoint and show that it is not relevant for slu7-depletion. This is as expected if the defect is in nuclear positioning. They could

test other checkpoint pathways that would monitor nuclear positioning in budding yeasts. Perhaps they have considered this: Bub2, Bfa1, Tem1, Lte1 mutants? I don't think this experiment is essential for publication, but it could strongly support their model.

Response: We appreciate the comment on other checkpoints operating during mitosis. However, we have not done these experiments to examine role of components that arrest mitosis (Bub2, Tem1 etc.) in response to spindle or kinetochore damage. We hope the reviewer appreciates that this line of work would require the generation of *bub2Δ* strain and extensive characterization for their role in checkpoint in Cryptococcus before it can be brought into strains compromised for Slu7.

Minor comments:

1. in Figure 3, Dyn1-GFP is imaged and in many of the cells in which Slu7 is depleted, nothing (or very little) can be seen. It is later argued that this is an indirect effect, due to defects in Pac1 and associated functions. Have the authors attempted a Dynein western blot (the 3xGFP tag should be quite sensitive)? It would be good to demonstrate that the Dynein motor complex hasn't simply fallen apart and Dynein been degraded in the slu7-depletion.

Response: A study in *S. cerevisiae* has reported the dynein expression does not change in *pac1Δ* cells (Lee et al., 2003). Since the molecular weight of CnnDYN1 along with the tag is 630kDa, we did attempt the very challenging experiment of western blot to check for the expression levels this very large protein in wildtype and *slu7kd* cells. Based on the reviewer's suggestion, we have attempted dot blot of protein lysates from wild type and from *slu7kd* cells probed with anti GFP antibody for estimating DYN-GFP levels. Untagged WT H99 strain was used as negative control. The same blot was stripped and re-probed for PSTAIRE which served as a loading control. This experiment revealed that dynein levels are same in both wildtype and *slu7kd* cells.

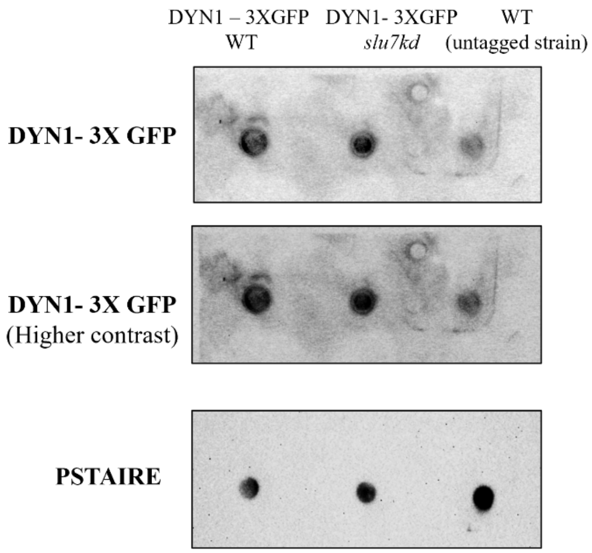

2. in Figure 7: have any intronless genes been tested for rescue of the post-mitotic delay/arrest? This is not necessary for publication, but if any have been tested already, they could be listed here.

Response: We have not tested intronless genes for their role in the rescue of post mitotic delay/arrest. From the RNA seq data, we observed that most of the genes involved in mitotic exit network (MEN) and cytokinesis were highly expressed in *slu7kd* cells as compared to the wildtype indicating and indirect role for Slu7 in their expression level. So, we had validated three candidates MOB2, CDC12 and DBF2 by qRT PCR (Supplementary 7.D) and found they were upregulated in *slu7kd* cells and hence speculate that deregulation of these transcript could contribute to the post mitotic arrest in *slu7kd.*

3. In SFig2C legend make it clear that these cells are HU arrested at time zero. Are the cells in glucose or galactose during HU treatment.?

Response: We regret the lack of clarity in the legend and the required details have been added. The cells were initially grown in non-permissive media for 2 hours to deplete Slu7 and then HU was added to the non-permissive media and the cell were allowed to grow for 4 hours.

4. in SFig4, the TBZ sensitivity isn't very convincing as the slu7kd strain is struggling to grow at all on YPD.

Response: We agree with the reviewer comment on the growth of *slu7kd* cells on media YPD containing TBZ. TBZ may exacerbate the defective mitosis of Slu7 depleted cells, however whether it pertains only to mitosis or any cellular processes where microtubules are involved requires further investigation.

In SFig5 legend the volcano plot needs to be better explained. What are the dashed lines etc. ?

Response: We regret missing these details on the volcano plot which has now been added to the legend.

Reviewer #3

We appreciate the views that our work provides strong evidence to support out conclusions that *Cryptococcus neoformans* Slu7 controls mitotic progression by efficient splicing of cell cycle regulators and cytoskeletal elements. We have taken all comments of the reviewer into account to revise our manuscript with additional data, and by improving the presentation. The key additional data are summarized below.

Major comments:

1) The authors claimed that CnSlu7 is the most divergent among the fungal homologs and closer to its human counterpart (Fig. 1A, Supplementary Fig 1A).
-Just based on the phylogenetic tree including limited members, as in Supplementary Fig. 1, it cannot be concluded that CnSlu7 is closer to its human counterpart since the basidiomycete yeast such as C. neoformans itself is more closely positions to humans compared to the ascomycete yeasts S. cerevisiae and Sch. pombe in phylogenetic tree analysis. It is strongly recommended to include other fungal species from the Basidiomycota, such as Ustilago maydis, in phylogenetic analysis in Supplementary Fig. 1.
- Conservation analysis among diverse eukaryotes is more meaningful data that the conservation withing the fungi group, so that it is recommended that the data of Fig. 1 A would be replaced with the revised Supplementary Fig 1.
-The analysis data on amino acid identities among Slu7 homologues should be presented to support the claim.

Response: We agree with the reviewer that our data would be better served by an improved analysis of the phylogenetic relationship between various Slu7 homologs. We have therefore reconstructed the phylogenetic tree by including other fungal groups. This is presented here and also in the revised manuscript Supplementary Figure 1A. These data too, show that *Cryptococcus* (*deneoformans and neoformans*) Slu7 is the most diverged among its homologs from various fungal species with its closest homologs being other pathogens *Puccinia graminis* and *Ustilago maydis*.

2) Despite that CnSlu7 is the main key subject, the comparative analysis of CnSlu7 to the previously reported Slu7 homologues, in the aspect of functional domain organization, is not provided in the present manuscript.
- It was reported that Slu7 contains the four motifs that control its cellular localization and canonical function as a splicing factor, such as a nuclear location signal, a zinc knuckle motif, four stretches of leucine repeats and a lysine-rich domain. Notably, human Slu7 protein is 204 amino acids longer than S. cerevisiae homolog with only 24% identity in the zinc knuckle motif (Molecular Biology of the Cell Vol. 15, 3782-3795). Thus, it is strongly recommended to provide additional information on the conserved and diverged features of CnSlu7 compared to other Slu7 homologs as a part of revised Figure.

Response: The multiple sequence alignment of Cryptococcus neoformans Slu7 with its fungal and higher eukaryote homologs such as human Slu7 and plant Slu7 proteins revealed that only the CCHC zinc finger motif is highly conserved. We do not detect conservation in the nuclear

localization signal, stretch of leucine repeats and lysine rich domain except for leucine 3 stretch near the C terminal. This additional information is presented in revised Figure 1A.

3) The manuscript clearly demonstrated that one of key targets of Slu7-mediated splicing is PAC1 in C. neoformans. Considering, Pac1 is also conserved from S. cerevisiae to human, it could be speculated that the defect of Slu7 can affect nuclear migration in other fungal species and human cells by inefficient splicing of PAC1, despite striking differences in their nuclear position during cell division. Please discuss this possibility or provide the qRT-PCR analysis data of PAC1 homologs in the available fungal Slu7 mutant strains.

Response: Cell cycle arrest phenotypes of splicing factor mutants (studied largely in budding and fission yeast) results from inefficient pre-mRNA splicing of cell cycle-related genes. Slu7 is a well characterized second step splicing factor in *S. cerevisiae* where *in vitro* splicing assays with ACT1 minigene transcripts with a modified single intron showed ScSlu7 is dispensable for splicing when the branchpoint to 3'SS distance is less than seven nucleotides in the mini transcript (Brys and Schwer, 1996). In fission yeast we reported the effects of metabolic depletion of Slu7, which is an essential gene (Banerjee et al., 2013) and showed unexpectedly that in addition to BrP to 3'SS distance new intronic features contributors of dependency of fission yeast intron containing transcripts on Slu7 functions. The work also showed in multi-intronic transcripts its role is intron-specific and thus the candidate gene/ transcript is likely to be to dependent on Slu7 by virtue of the intronic features and not its biological function. In this study a splicing dependent role of CnSlu7 in cell cycle progression is investigated where based on a strong nuclear mis-positioning phenotype we narrowed on PAC1 transcripts as one of targets. We show PAC1, encoding a cytoskeletal factor, has introns dependent on CnSlu7 for efficient splicing and show partial rescue of nuclear position in strain complemented with expression of an intronless PAC1 gene. In this scenario, while it is likely that in other species where PAC1 exon-introns nucleotide sequences are similar to that in Cryptococcus a role for Slu7 may be predicted, for validation by other experimentalists.

Interestingly, *PAC1* in *S. cerevisiae* is an intronless gene and its homolog is not annotated in *S. pombe.* In human cell lines, knockdown of Slu7 by siRNA resulted in metaphase arrest by inefficient splicing of soronin – which is crucial in sister chromatid cohesion and correct spindle assembly, according to recent research in human cell lines (Jiménez et al., 2019).

Hence the roles of splicing factor in cell cycle is through splicing of targets involved in cell cycle wherein the targets regulated by splicing factor may or may not be conserved in other species.

Minor comments:

General points

1) Provide information on the marker sizes in the data of qRT-PCR analysis presented in Figures 5 and 6, and Supplementary Fig 2A.

Response: We regret the omission of this technical data and have corrected the same by providing the marker sizes in all the figures.

2) Please unify the format of gene names. Some genes were written with superscript of "+", such as CLN1+ and PAC1+ in Fig. 4. What does "+" mean in the gene names?

Response: We have taken the suggestion to carefully review the nomenclature of genes and their expressed transcripts as is typical for Cryptococcus neoformans. To depict the wildtype form of transcript we had used +. Thus CLN1+ was used to denote Cyclin 1 cellular transcript from expressed from its own locus without any modification of promoter or the intronic features.

3) Supplementary Figure 1 C: Please correct "Slu7KD" 6 hrs YPD to "slu7kd" 6 hrs YPD.

Response: This error has been corrected.

4) Supplementary Figure 2A: What do "mRNA" and "No RT29X/", respectively, indicate?

Response: The mRNA indicates the spliced form across any intron after intron is spliced out, so denotes exon-exon sequences in the mRNA. The reactions marked as "No RT 29 X" denote semi- quantitative PCR performed on DNase treated RNA sample, without reverse transcription to generate the cDNA. These reactions were done to confirm that there is no genomic DNA present in the RNA sample used for reverse transcription reaction of the cellular transcripts. Some of these details are now included in the Supp Fig 2A legend.

5) Supplementary Figure 4C: Please provide brief explanation in the text on why the authors employed mad2Δ slu7kd cells.

Response: In Page 8, line 6, we had provided the rationale for generating and studying mad2Δ slu7kd strain. This is recapitulated below:

"To investigate whether Slu7 knockdown triggers the activation of spindle assembly checkpoint (SAC), we generated a strain with conditional slu7kd in cells with mad2Δ allele and the GFP-H4 nuclear marker."

6) Supplementary Figure 6D legend: Please correct the description of "slu7kd SH:Slu7 FL" from "expressing intronless PAC1" to "expressing full length of SLU7".

Response: The error in the legend is regretted and this has been corrected.

7) Supplementary Figure 7D: The authors confirmed that MOB2, CDC12, and DFB1 were

expressed at higher levels in slu7kd when compared to wildtype. Please briefly explain in the text why the expression level of these genes in slu7kd was mentioned.

Response: *slu7kd* cells expressing intronless Pac1 arrest post nuclear division. Revisiting our transcriptomic data, we found that genes involved in mitosis exit network and cytokinesis, such as DFB1, MOB2, CDC12, BUD4, and CHS2, were deregulated in *slu7kd* when compared to wildtype. We confirmed the same by performing qRT PCRs for three candidates, MOB2, DBF1 and CDC12 and that these transcript were expressed at high levels in knockdown when compared to wildtype.

8) The species name should be written as abbreviation after the first mention. For example, please correct Cryptococcus neoformans to C. neoformans throughout manuscript.

Response: The suggestion is well taken, and the required edits have been made throughout the text.

9) Please unify the format of paper titles listed in References.

Response: This formatting error is regretted and corrected to have all references in a single format.

10) No page information for Hoffmann et al (2010) in References.

Response: This omission is corrected.

11) Update the information on the published journal of Chatterjee et al. (2021) in References.

Response: This omission is regretted and is now corrected.

12) Information on the authors, title, published journal and pages should be provided for the papers (Yadav and Sanyal, 2018; Sridhar et al., 2021) in Supplementary Table 1, which were not included in the main Reference list.

Response: The references are now added to the main list.

References used for addressing the reviewer's comments:

1. Chung DKC, Chan JNY, Strecker J, Zhang W, Ebrahimi-Ardebili S, Lu T, Abraham KJ, Durocher D, Mekhail K (2015) Perinuclear tethers license telomeric DSBs for a broad kinesin- and NPC-dependent DNA repair process. *Nat Commun* 6. doi:10.1038/NCOMMS8742.

2. Jiménez M, Urtasun R, Elizalde M, Azkona M, Latasa MU, Uriarte I, Arechederra M, Alignani D, Bárcena-Varela M, Alvarez-Sola G *et al* (2019) Splicing events in the control of genome integrity: Role of SLU7 and truncated SRSF3 proteins. *Nucleic Acids Res* 47: 3450–3466. doi:10.1093/nar/gkz014.
3. Laflamme G, Sim S, Leary A, Pascariu M, Vogel J, D'Amours D (2019) Interphase Microtubules Safeguard Mitotic Progression by Suppressing an Aurora B-Dependent Arrest Induced by DNA Replication Stress. *Cell Rep* 26: 2875-2889.e3. doi:10.1016/J.CELREP.2019.02.051.
4. Lawrimore J, Barry TM, Barry RM, York AC, Friedman B, Cook DM, Akialis K, Tyler J, Vasquez P, Yeh E *et al* (2017) Microtubule dynamics drive enhanced chromatin motion and mobilize telomeres in response to DNA damage. *Mol Biol Cell* 28: 1701–1711. doi:10.1091/MBC.E16-12-0846.
5. Lee WL, Oberle JR, Cooper JA (2003) The role of the lissencephaly protein Pac1 during nuclear migration in budding yeast. *J Cell Biol*. doi:10.1083/jcb.200209022.
6. Lottersberger F, Karssemeijer RA, Dimitrova N, De Lange T (2015) 53BP1 and the LINC Complex Promote Microtubule-Dependent DSB Mobility and DNA Repair. *Cell* 163: 880–893. doi:10.1016/J.CELL.2015.09.057.
7. Oshidari R, Strecker J, Chung DKC, Abraham KJ, Chan JNY, Damaren CJ, Mekhail K (2018) Nuclear microtubule filaments mediate non-linear directional motion of chromatin and promote DNA repair. *Nat Commun* 9. doi:10.1038/S41467-018-05009-7.
8. Varshney N, Som S, Chatterjee S, Sridhar S, Bhattacharyya D, Paul R, Sanyal K (2019) Spatio-temporal regulation of nuclear division by Aurora B kinase Ipl1 in Cryptococcus neoformans. *PLoS Genet* 15. doi:10.1371/journal.pgen.1007959.
9. Wu G, Zhou L, Khidr L, Guo XE, Kim W, Lee YM, Krasieva T, Chen PL (2008) A novel role of the chromokinesin Kif4A in DNA damage response. *Cell Cycle* 7: 2013–2020. doi:10.4161/CC.7.13.6130.
